# The Impact of Body Mass Index on Morbidity and Mortality in Iraqi Patients With Heart Failure

**DOI:** 10.7759/cureus.71043

**Published:** 2024-10-07

**Authors:** Abdulameer Abdulhameed, Mohammed Allami, Hassan M Dubais

**Affiliations:** 1 Department of Medicine, College of Medicine, University of Basrah, Basrah, IRQ; 2 Internal Medicine, College of Medicine, University of Duhok, Duhok, IRQ; 3 Internal Medicine, Al Qurnah Hospital, Basrah, IRQ

**Keywords:** body mass index: bmi, ejection fraction, heart failure, iraq, obesity

## Abstract

Background: Obesity and heart failure (HF) are increasingly significant contributors to illness and death worldwide. While obesity appears to increase the risk of HF, it may paradoxically improve survival. This study aimed to investigate the impact of body mass index (BMI) on the mortality and morbidity of patients with HF.

Methodology: A total of 122 patients including females (n=39) and males (n=83) diagnosed with HF were admitted to two cardiac units in Basrah, Iraq. The diagnosis was made based on Framingham Heart Failure Diagnostic Criteria. The BMI, baseline hemodynamics, and medical history were recorded, while the etiology and severity of HF were assessed at enrollment. The patients were followed up prospectively for hospital admission, and survival after one year of enrollment.

Results: The mean age of patients with HF was 62.7 years (SD 10.25). They included based on BMI categories 49.2% of normal/underweight individuals, while 50.8% were overweight/obese. The most frequent etiology was ischemic heart disease in 63.9%. Echocardiography revealed that the prevalence of left ventricular ejection fraction (LVEF) < 50% was 62.29%. It was observed that obese/overweight patients were more likely to have diastolic HF (P=0.001), and more severe disease (P=0.014), and were more likely to be alive at the one-year mark (P=0.001) than underweight/normal ones. Furthermore, the underweight had the least favorable outcome than any of the other five BMI categories (P<0.0001). Moreover, obese/overweight individuals had higher hospitalization rates in the first six months than normal/underweight, though this was insignificant (P=0.15).

Conclusions: It appears that among Iraqis with heart failure, those who are overweight or obese had better outcomes at one year compared to normal or underweight. Further studies on a larger number of HF patients, and utilizing additional anthropometric indices and cardiorespiratory fitness to validate the observation of this study are warranted.

## Introduction

Obesity and heart failure (HF) are major contributors to illness and death globally. Despite advances in medical and surgical treatments, the yearly incidence of HF continues to rise [1]. In developed countries, the prevalence of congestive heart failure (CHF) is around 1-2% of the population. Every year, around 1.4 million new cases of HF develop due to various reasons, including high blood pressure, coronary artery disease, heart muscle inflammation, and damage from rheumatic fever [2]. Several studies have confirmed a higher risk of CHF among overweight and obese individuals. This risk increases progressively with Body Mass Index (BMI), with men experiencing 5%, and women 7% greater chance of developing CHF per BMI point increase [3]. Obesity's association with HF is attributed to multiple factors, including higher rates of hypertension, increased incidence of diabetes mellitus, and dyslipidemia [4]. Moreover, obesity is associated with a variety of hemodynamic alterations that may predispose to left and right heart failure, including increased preload and afterload due to hyperdynamic circulation, chronic volume overload, and increased peripheral resistance [5]. Research indicates that overweight individuals with mild sleep apnea are more prone to developing metabolic syndrome, hypertension, prediabetes, and cholesterol abnormalities, particularly high triglyceride levels [6]. Obesity is associated with the release of substances into the bloodstream that can potentially lead to plaque rupture and heart attacks. While obesity is generally correlated to higher rates of mortality in the general population, several studies have surprisingly shown lower mortality rates among overweight and obese patients with established heart failure, a phenomenon referred to as the "obesity paradox" [7-9].

There is a limited number of studies from our part of the world on the relevance of BMI to mortality and morbidity in patients with heart failure. Accordingly, the current study was initiated to address this issue.

## Materials and methods

In this observational prospective study, a total of 122 patients diagnosed with HF were recruited. The patients were recruited as they attended the Cardiac Care Unit and medical wards in Al Fayhaa General and Al Sadr Teaching Hospitals in Al-Basrah, Iraq in the period between April 2017 and December 2018.

All recruited patients had a complete history, and full physical exam at the time of enrolment. Their medical records were also thoroughly reviewed. Moreover, all patients had their ECG, and transthoracic echocardiography performed at the time of enrolment, and whenever that is required in their follow-up period. The standard two-dimensional and M Mode echocardiography measurements of the cardiac chambers were taken. The diagnosis of HF was made according to Framingham Criteria as detailed elsewhere [10]. On the other hand, the patients were categorized using the New York Heart Association (NYHA) functional classification of HF severity [10] and as follows: Stage I: Cardiac disease without limitation of physical activity. Ordinary physical activity is not associated with undue fatigue, dyspnea or chest pain; Stage II: Physical activity is slightly limited. Patients are comfortable at rest. Ordinary physical activity may result in fatigue, palpitation, dyspnea, or angina; Stage III: Physical activity is markedly limited. Patients are still comfortable at rest, but less than ordinary physical activity causes fatigue, palpitation, dyspnea, or chest pain; Stage IV: Inability to carry on any physical activity without discomfort, and symptoms of heart failure or angina are present at rest.

The BMI was calculated for each patient based on their weight and height at enrollment, and patients were classified into six categories: underweight (<18.5 kg/m2), normal weight (18.5-24.9 kg/m2), overweight (25-29.9 kg/m2), Class I obesity (30-34.9 kg/m2), Class II obesity (35-39.9 kg/m2), and Class III obesity (>40 kg/m2) [11].

Furthermore, and based on clinical, echocardiography, and ECG findings, the patients were classified according to HF etiology into the following categories: ischemic heart disease, dilated cardiomyopathy, toxic heart disease, hypertensive heart disease, and valvulopathy.

To track patients' outcomes, researchers followed them up, and documented their clinical status at the end of one year, checking whether they were alive, and if so, their records were retrieved and admission history recorded.

The research was approved by the ethical committee review board at the College of Medicine, University of Basrah, Iraq (ref. number: 0304-8067-2017), and informed written consent was obtained from all enrolled patients.

Statistical analyses were performed using SPSS version 22.0 (IBM Corp., Armonk, NY, USA). Mean and Standard Deviation (SD) were used to describe continuous variables, while number (percentage) was used for categorical variables. Chi-square test was used to compare categorical variables, while Student's t-test was used to compare continuous ones. A value of P<0.05 was considered significant.

## Results

One hundred and twenty-two HF patients met the inclusion criteria for this study and they consisted of 83 males and 39 females. The distribution of patients according to their BMI categories is outlined in Table 1, with 60 patients (49.2%) being underweight or normal, while 62 patients were overweight or obese. In relevance to the etiology of HF, the most frequent was ischemic heart disease in 78 cases (63.9%), followed by valvulopathy in 21 cases (17.2%), dilated cardiomyopathy in 14 cases (11.5%), hypertensive heart disease in another eight cases (6.6%), while the least frequent was toxic heart disease in one case (0.8%). Further baseline and hemodynamic characteristics of the enrollees are detailed in Table 1.

**Table 1:** Baseline and some hemodynamic characteristics in 122 heart failure patients LBBB: Left bundle branch block, LVEF: Left ventricular ejection fraction

Parameter	Mean (SD)
Age (years)	62.80 (10.26)
Weight (kg)	74.15 (16.72)
Height (cm)	170.23 (7.38)
Heart rate (beat/minute)	81.46 (11.96)
Systolic blood pressure (mmHg)	123.03 (15.91)
Diastolic blood pressure (mmHg)	84.47 (12.79)
BMI (kg/m2)	25.78 (6.43)
	Number (%)
BMI category	
Underweight	14 (11.5)
Normal weight	46 (37.7)
Overweight	33 (27)
Obese Class I	17 (13.9)
Obese Class II	8 (6.6)
Obese Class III	4 (3.3)
Baseline hemodynamics	
LVEF > 50%	46 (37.7)
LVEF < 50%	76 (62.29)
LBBB	47 (38.5)
No LBBB	75 (61.47)
Admissions	
Within 6 months of enrollment	71 (58.2)
More than 6 months	51 (41.8)

Table 2 shows that overweight and obese patients were significantly more likely to have diastolic heart failure, while normal and underweight patients were more likely to have systolic heart failure (P=0.001). As per NYHA functional HF categories, obese/overweight patients were more likely to have classes III and II, while underweight/normal weight patients had classes II and I as the most frequent, an observation which was significant (P=0.014).

**Table 2:** Body mass index in relation to type and severity of heart failure (HF), frequency of hospitalization, and one-year mortality rate NYHA: New York Heart Association

Parameter	BMI category		P Value
	Obese/overweight (n=62)	Normal/underweight (n=60)	
Type of heart failure			0.001
Diastolic HF	35 (56.45)	11 (18.33)	
Systolic HF	27 (43.55)	49 (81.67)	
NYHA classification			0.014
I	8 (12.90)	17 (28.33)	
II	22 (35.48)	28 (46.67)	
III	25 (40.32)	10 (16.67)	
IV	7 (11.29)	5 (8.33)	
Early versus later hospitalization			0.15
Within the first 6 months	40 (64.52)	31 (51.67)	
More than 6 months	22 (35.48)	29 (48.33)	
One year mortality			0.001
Alive	57 (91.94)	33 (55.0)	
Dead	5 (8.06)	27 (45.00)	

There was no significant difference in hospitalization in the first six months, compared to that beyond this timeframe between underweight/normal patients and overweight/obese (P=0.15), while a significantly lower proportion of underweight/normal BMI were alive at the one-year follow-up mark, compared to overweight/obese HF patients (P=0.001) (Table 2).

Table 3 shows that the male patients were more likely to be overweight or class I obese while females were more likely to be class II/III obese (P=0.03). Patients with left ventricular ejection fraction (LVEF) <50% were more likely to be of normal weight (P=0.001). On the other hand, there was no statistically significant association between BMI and the etiology of HF.

**Table 3:** Some baseline characteristics of 122 heart failure patients categorized into the six BMI categories LBBB: Left bundle branch block, LVEF: Left ventricular ejection fraction

Variables (frequency)	BMI						P value
	Underweight (n=14)	Normal weight (n=46)	Overweight (n=33)	Class I obesity (n=17)	Class II obesity (n=8)	Class III obesity (n=4)	
Male	9 (11.3)	29 (36.3)	26 (32.5)	13 (16.3)	2 (2.5)	1 (1.3)	0.03
Female	5 (11.9)	17 (40.5)	7 (16.7)	4 (9.5)	6 (14.3)	3 (7.1)	
LVEF>50%	6 (13.0)	5 (10.9)	13 (28.3)	14 (30.4)	6 (13.0)	2 (4.3)	0.001
LVEF<50%	8 (10.5)	41 (53.9)	20 (26.3)	3 (3.9)	2 (2.6)	2 (2.6)	
LBBB	4 (8.5)	19 (40.4)	6 (12.8)	12 (25.5)	3 (6.4)	3 (6.4)	0.014
Etiology							0.267
Ischemic heart disease	9 (11.5)	31 (39.7)	16 (20.5)	12 (15.4)	6 (7.7)	4 (5.1)	
Dilated cardiomyopathy	1 (7.1)	5 (35.7)	4 (28.6)	3 (21.4)	1 (7.1)	0 (0.0)	
Hypertensive heart disease	0 (0.0)	4 (50.0)	2 (25.0)	2 (25.0)	0 (0.0)	0 (0.0)	
Toxic heart disease	1 (100)	0 (0.0)	0 (0.0)	0 (0.0)	0 (0.0)	0 (0.0)	
Valvulopathy	3 (14.3)	6 (28.6)	11 (52.4)	0 (0.0)	1 (4.8)	0 (0.0)	

After one year's follow-up, death was reported in 32 patients. The recorded causes of death were heart failure in 15, myocardial infarction in seven, other cardiovascular causes in four, while stroke and unknown causes were recorded in three patients each. The study showed that the underweight had the highest death rate after one year's follow-up than any other category, with those of normal weight coming next in frequency far exceeding those from the other four BMI categories (p<0.0001; Table 4). Moreover, it was found that those who were alive at the one-year mark had a mean BMI (SD) of 27.34 (5.9) kg/m2, which was significantly higher than non-survivors (BMI mean (SD) = 21.36 (5.6) kg/m2) (P=0.001).

**Table 4:** Correlation of mortality rate in one year and body mass index in heart failure patients

Survival rate in 1 year	Body mass index (kg/m2)						P value
	Under weight (n=14)	Normal weight (n=46)	Overweight (n=33)	Class I obesity (n=17)	Class II obesity (n=8)	Class III or morbid obesity (n=4)	
Alive	5 (35.7)	28 (60.9)	30 (90.9)	16 (94.1	7 (87.5)	4 (100)	<0.0001
Dead	9 (64.3)	18 (39.1)	3 (9.1)	1 (5.9)	1 (12.5)	0 (0.0)	

## Discussion

Obesity is recognized as a significant risk factor for heart failure in both men and women, and its prevalence has been increasing globally since the 1980s. Paradoxically, several studies have indicated better outcomes in obese patients with heart failure [9]. The mean age of patients in our study was 62.8 years, and included 32% females. This finding is in line with an earlier large multinational study on heart failure which reported a mean age of 65 years with 35% females [8].

In the current study, the most common heart failure etiology was ischemic heart disease, followed by valvular heart disease, which is consistent with earlier studies that reported from previous studies on heart failure [12,13].

In the current study, females were significantly more likely to be in the highest two BMI categories (Obese II & III) than males, which is consistent with an earlier study on more than 4000 heart failure patients from Europe [14]. Moreover, it was found diastolic heart failure was significantly more likely in obese/overweight as compared to normal/underweight individuals. The latter observation is consistent with multiple earlier studies based on echocardiographic and radionuclide studies [15,16].

Despite the fact that overweight/obese individuals were significantly more symptomatic as per NYHA heart failure categorization than normal/underweight patients, it was documented that overweight/obese patients were more likely to be alive at one year's follow-up than underweight/normal individuals. Such an observation is not unique to the current study, and several studies have revealed that obese people have better prospects of survival than their leaner counterparts [7,17,18]. Similarly, Horwich et al. reported that underweight followed by normal-weight individuals had the worst, while obese and overweight individuals had the best prognosis [9]. Likewise, researchers of the CHARM study, which is a multinational double-blind study reporting on about 7600 heart failure patients, revealed that lower BMI was associated with a higher risk of death from all causes, and similar to the current study also revealed that the hospitalization rate was not significantly different [8]. Furthermore, a meta-analysis including more than 28000 HF patients reported that overweight individuals had a 16% reduction in all-cause mortality, while obese individuals had a 33% respective reduction when compared to normal-weight individuals [19]. Similarly, a systematic review of six studies demonstrated that all-cause and cardiovascular mortality were higher in underweight patients with chronic HF compared to those who were overweight [20]. Moreover, it appears that this favorable effect of obesity extends to acute decompensated HF, and the ADHERE advisory committee and investigators, based on more than 108,000 patients, concluded that for each five units increase in BMI there is a 10% decrease in mortality [21].

The current study found that LVEF was significantly more likely to be in the <50% category in normal/underweight categories than overweight/obese categories. Such an observation may be linked to higher mortality in these BMI categories, since low LVEF has been linked to poor prognosis in HF [22]. Our findings are consistent with I-PRESERVE study observations, including more than 4000 HF patients, where LVEF was higher in those with higher BMI [14], though this study focused on HF with preserved LVEF.

The precise mechanism behind the obesity paradox remains elusive and continues to challenge researchers seeking explanations. Overweight and obese individuals often develop HF secondary to conditions like hypertension and cardiovascular disease, which are exacerbated by excessive weight and can directly lead to left ventricular dysfunction. It has been hypothesized that the poorer prognosis observed in underweight individuals with HF may stem from the less frequent occurrence of HF due to hypertension and typical coronary artery disease, possibly indicating a condition with more severe outcomes when it does occur. Additionally, obese patients tend to present at a younger age and exhibit lower levels of circulating natriuretic peptides, which are associated with less severe disease presentation and potentially better prognosis [23,24]. Furthermore, overweight and obese patients often have higher lipid profiles, which may contribute to improved mortality in HF by binding circulating endotoxins and potentially mitigating their harmful effects [25]. These factors underscore the complex interplay of physiological and pathological processes that influence outcomes in HF patients of varying BMI categories.

Other researchers suggest that obese and overweight patients with chronic HF have an overactive nervous system (sympathetic) and hormone system (renin-angiotensin-aldosterone system (RAAS)). Since most HF patients also have high blood pressure (hypertension), stronger medications like beta-blockers, RAAS inhibitors, and aldosterone antagonists might be beneficial [26].

Another plausible explanation for these observations is that HF represents a catabolic state, where the body's metabolic processes are disrupted. Obese individuals may possess greater metabolic reserves, enabling them to better withstand the physiological stresses of HF and potentially leading to better survival outcomes. In contrast, cachexia, characterized by severe weight loss and muscle wasting, is associated with a poorer prognosis in HF patients [9].

Another perspective suggests that obesity imposes a form of "compulsory exercise" on individuals, as they may exert greater effort due to their higher body mass. In the context of heart failure, where exercise is often recommended for its cardiovascular benefits, this increased physical demand in obese individuals could potentially contribute to their better prognosis [27].

Despite the evidence supporting the “obesity paradox” by the current and earlier studies, some recent studies have suggested that anthropometric indices such as waist-to-height ratio may be more reflective of central obesity than BMI [28], while others suggest that weight is only part of the obesity paradox and that cardiorespiratory fitness may be a major factor influencing the paradox [29]. Moreover, a recent trial suggested that while the obesity paradox (based on BMI) was evident in those with reduced LVEF HF, it was eliminated by adjusting for other prognostic variables [30].

Limitations of the study

The limitations of the current study include the fact that we only used BMI to categorize obesity, and we did not include other anthropometric indices, or cardiorespiratory fitness as additional assessment tools, in addition to the relatively limited number of patients enrolled.

## Conclusions

This study has shown that among Iraqi HF patients, those who were overweight and obese had much better outcomes compared to those who were normal or underweight. Further studies including the use of anthropometric indices and adjusting for other confounders on a larger number of participants are warranted. Such studies would be important to aid in personalizing treatment plans for obese patients with heart failure in this part of the world.
